# Beyond Bruton’s tyrosine kinase inhibitors in mantle cell lymphoma: bispecific antibodies, antibody–drug conjugates, CAR T-cells, and novel agents

**DOI:** 10.1186/s13045-023-01496-4

**Published:** 2023-08-25

**Authors:** Neeraj Jain, Mukesh Mamgain, Sayan Mullick Chowdhury, Udita Jindal, Isha Sharma, Lalit Sehgal, Narendranath Epperla

**Affiliations:** 1 Division of Cancer Biology, CSIR-Central Drug Research Institute, Lucknow, Uttar Pradesh India; 2 Academy of Scientific and Innovative Research, Ghaziabad, Uttar Pradesh 201002 India; 3 Department of Medical Oncology and Hematology, All India Institute of Medical Sciences, Rishikesh, India; 4 Division of Hematology, Department of Medicine, Arthur G. James Cancer Hospital and Richard J. Solove Research Institute, The Ohio State University, Columbus, OH USA; 5 The Ohio State University Comprehensive Cancer Center, Suite 7198, 2121 Kenny Rd, Columbus, OH 43221 USA

**Keywords:** BTK inhibitor resistance, Mantle cell lymphoma, CAR T cell therapies, Bispecific antibodies, Antibody–drug conjugates

## Abstract

Mantle cell lymphoma is a B cell non-Hodgkin lymphoma (NHL), representing 2–6% of all NHLs and characterized by overexpression of cyclin D1. The last decade has seen the development of many novel treatment approaches in MCL, most notably the class of Bruton's tyrosine kinase inhibitors (BTKi). BTKi has shown excellent outcomes for patients with relapsed or refractory MCL and is now being studied in the first-line setting. However, patients eventually progress on BTKi due to the development of resistance. Additionally, there is an alteration in the tumor microenvironment in these patients with varying biological and therapeutic implications. Hence, it is necessary to explore novel therapeutic strategies that can be effective in those who progressed on BTKi or potentially circumvent resistance. In this review, we provide a brief overview of BTKi, then discuss the various mechanisms of BTK resistance including the role of genetic alteration, cancer stem cells, tumor microenvironment, and adaptive reprogramming bypassing the effect of BTK inhibition, and then provide a comprehensive review of current and emerging therapeutic options beyond BTKi including novel agents, CAR T cells, bispecific antibodies, and antibody–drug conjugates.

## Background

Mantle cell lymphoma (MCL) is a subtype of B cell non-Hodgkin lymphoma (NHL) characterized by overexpression of CCND1 and translocation t(11:14)(q13;q32) [1]. The most common type of MCL originates from mature B cells and is often found to become unstable and aggressive through accumulating mutations in genes related to cell cycle regulation, such as the DNA damage response pathway. They are often found to express SOX11 [2] and carry little to no immunoglobulin heavy variable (IGHV) somatic mutations [2] and include classical, blastoid, and pleomorphic variants of MCL. The second indolent subtype (10–15% cases, leukemic non-nodal variant) is less aggressive, carries IGHV somatic hypermutations [1], and is genetically stable with low to no SOX11 expression. Patients can potentially have asymptomatic disease with this subtype for extended periods [3]. Recently, the diagnosis-to-treatment interval (DTI) was shown to be prognostic patients with newly diagnosed MCL, wherein patients with short DTI (DTI ≤ 14 days) had worse outcomes and was strongly associated with adverse clinical factors [4]. While the outcomes of MCL have been conventionally poor, there has been improvement in survival in the past decade owing to the advent of novel therapies [5].

The treatment of MCL in the frontline setting largely relies on patient-specific factors such as age, overall performance status, and underlying co-morbidities. For the young, transplant-eligible patient, treatment generally consists of induction chemotherapy, consolidation with an autologous stem cell transplantation, and maintenance with rituximab for about three years. For induction chemotherapy, no specific chemotherapy regimen has been firmly established as the standard of care, and the treatment regimen used is variable based on the institution or physician's practice. However, it is generally accepted that the regimen should contain rituximab and cytarabine. Less toxic chemotherapy treatments are given for patients unfit for intensive chemotherapy, such as bendamustine/rituximab (BR) or R-CHOP (rituximab, cyclophosphamide, doxorubicin, vincristine, and prednisone), with or without maintenance rituximab. However, patients eventually progress following frontline therapy, so establishing effective treatments for relapsed/refractory (R/R) MCL is important. In the past decade, BTK inhibitors (BTKi) have revolutionized the management of patients with R/R MCL. However, a significant proportion of patients eventually progress with poor post-BTKi relapse outcomes.

This review article focuses on three main aspects: (1) discuss the current BTKis approved for clinical use in the USA, (2) detail the various mechanisms of BTK resistance including the role of genetic alteration, cancer stem cells, tumor microenvironment, and adaptive reprogramming bypassing the effect of BTK inhibition, and (3) current and emerging therapeutic strategies beyond BTKi.

### BTKi in MCL

Currently, there are four BTKis approved for MCL therapy. They are ibrutinib, acalabrutinib, zanubrutinib (all covalent BTKis), and pirtobrutinib (only approved non-covalent BTKi) [6–10]. Table 1 summarizes the BTKi currently approved for MCL, the study that led to their FDA approval, and the potential adverse effects reported on these studies.

**Table 1:** FDA-approved BTKi in MCL

Name of approved BTKi	Type	Study design	Pivotal study*	ORR	CRR	Adverse effects
Ibrutinib	Covalent	Phase 2	Targeting BTK with ibrutinib in relapsed or refractory mantle cell lymphoma. *N Engl J Med* 369: 507–516	68%	21%	Diarrhea (50%), fatigue (41%), nausea (31%), peripheral edema (28%), dyspnea (27%), constipation (25%), upper respiratory tract infection (23%), vomiting (23%), and decreased appetite (21%)
Acalabrutinib	Covalent	Phase 2	Acalabrutinib in relapsed or refractory mantle cell lymphoma (ACE-LY-004): a single-arm, multicenter, Phase 2 trial. *Lancet* 2018; 391(10121):659–667	81%	40%	Headache (38%), diarrhea (31%), fatigue (27%), and myalgia (21%). Neutropenia (10%), anemia (9%), and pneumonia (5%)
Zanubrutinib	Covalent	Phase 2	Treatment of patients with relapsed or refractory mantle cell lymphoma with zanubrutinib, a selective inhibitor of Bruton’s tyrosine kinase. *Clin Cancer Res.* 2020;26(16):4216–4224	84%	69%	Neutropenia (48.8%), Leukopenia (34.9%), Thrombocytopenia (32.6%) Anemia (15.1%) URI (34.9%) Rash (33.7%) Hypokalemia (16.3%) Diarrhea (15.1%) Hypertension (15.1%) Lung infection (12.8%) ALT elevation (14%)
Pirtobrutinib	Non-covalent	Phase 2	Pirtobrutinib in relapsed or refractory B cell malignancies (BRUIN): a phase 1/2 study. Lancet. 2021 Mar 6;397(10277):892–901	51% in BTK pretreated and 82% in BTK naïve patients	13%	Fatigue (23%), diarrhea (19%), neutropenia (18%), and contusion (17%)

*BTKi* BTK inhibitors, *ORR* overall response rate, *CRR* complete response rate

*Study that led to FDA approval of the drug

### Resistance to BTKis and approaches for targeting mutant BTK

Although the first-generation BTKi (ibrutinib) has shown encouraging therapeutic effects in MCL, nearly one-third of treated patients have ultimately been found to develop primary intrinsic resistance. Additionally, acquired resistance developed in nearly all patients [11]. Furthermore, those patients who developed ibrutinib resistance have typically had dismal clinical outcomes, with median overall survival (OS) of ~ 6 to 8 months even after salvage treatment [9, 12–14]. The primary resistance is often associated with sustained activation of the PI3K-AKT pathway or other genetic alterations providing an alternative activation of B cell receptor (BCR) signaling [15]. In contrast, secondary resistance in MCL patients is found in patients harboring a point mutation in the BTK gene (*BTK*^*C481S*^*)* which reduces the binding affinity of covalent BTKis to BTK [16]. Second-generation BTKis such as acalabrutinib, tirabrutinib (ONO/GS-4059), spebrutinib (CC-292), and zanubrutinib (BGB-3111), which are typically more sensitive than ibrutinib, work by binding covalently and irreversibly to ATP binding region within the kinase domain of BTK at cysteine 481 position, and therefore, a point mutation in BTK (*BTK*^*C481S*^*)* can prevent the activity of these agents [17, 18]. In addition to the *BTK*^*C481S*^ mutation, the gain of function mutations in *PLCG2* (R665W, L845F, S707Y) has also been attributed to the secondary mechanism of ibrutinib resistance. However, *BTK*^*C481S*^ mutation is infrequent in practice, and mutations in *PLCG2* are typically not observed in MCL [19, 20]. In order to address resistance against first and second-generation covalent BTKis, third-generation non-covalent (reversible) BTKis (those could bind to both wild-type and *BTK*^*C481S*^) and proteolysis-targeting chimeras (PROTACs) targeting BTK have been designed, and many of them are being investigated in preclinical/clinical studies or already approved for use [21].

#### Non-covalent BTKi

Pirtobrutinib (LOXO-305) blocks the ATP binding site of BTK and, unlike ibrutinib, shows no direct interaction with C481. A recent study from Gomez et al. described pharmacologic, biophysical, and structural attributes that detailed and differentiated pirtobrutinib from the current covalent BTKi (ibrutinib, zanubrutinib, and acalabrutinib). This preclinical study demonstrated differential binding of pirtobrutinib to both *BTK* and *BTK*^*C481*^ substitution mutants that prevented BTK^Y551^ phosphorylation in the activation loop and inhibited BTK signaling in multiple B cell lymphoma cell lines and lymphoma xenograft tumor growth [22]. Pirtobrutinib is the first and only non-covalent reversible inhibitor that received FDA approval (27th January 2023) for R/R MCL patients. This is based on promising results in the BRUIN Phase 1/2 trial (NCT03740529) with an overall response rate (ORR) of 52% and complete response (CR) rate of 13% [23]. This study included 120 patients with MCL that were previously treated with a covalent BTKi—ibrutinib (67%), acalabrutinib (30%), and zanubrutinib (8%). Among these 120 MCL cases, 83% discontinued their last BTKi due to refractory or progressive disease. The trial data suggest that pirtobrutinib was well tolerated at all doses tested (the maximum tolerated dose was not reached). The updated data were presented in ASH 2022 meeting [24]. These data indicate that pirtobrutinib could be a potential treatment strategy to overcome covalent BTKi-associated resistance development in MCL. A phase 3 trial, BRUIN MCL-321 (NCT04662255) comparing pirtobrutinib monotherapy to the investigator's choice of covalent BTKi monotherapy (ibrutinib, acalabrutinib, or zanubrutinib) in MCL patients (*n* = 500) who received ≥ 1 prior line of systemic therapy that did not include a prior BTKi is currently ongoing [25].

Besides pirtobrutinib, other BTK-targeting non-covalent inhibitors are currently in the pipeline as well. Promising agents include fenebrutinib (GDC-0853) that inhibits BTK via forming hydrogen bonds with K430, M477, D539 of BTK and nemtabrutinib (MK-1026, formerly ARQ 531) that binds BTK via hydrogen bonds with E475, Y476 residues [26, 27]. These inhibitors have been tested in MCL, chronic lymphocytic leukemia (CLL), diffuse large B cell lymphoma (DLBCL), follicular lymphoma (FL), and other B cell malignancies [27, 28]. These agents have demonstrated acceptable safety profiles and efficacy against *BTK*^*C481S*^ mutant B cell malignancies in phase 1 clinical trials (NCT01991184, NCT03162536). However, only a few MCL cases have been included in these studies. As such, due to the lack of convincing data on these agents in the MCL population, further comments on the efficacy of these inhibitors in MCL cannot be made at this time.

#### BTK-PROTAC

PROTAC strategy is a novel approach that degrades a target of interest by bringing it to the proximity of E3 ubiquitin–proteasome ligase (Cereblon or Von Hippel–Lindau) through an attached linker. Several BTK-targeting PROTAC have been synthesized to overcome ibrutinib resistance, which could potentially target both the mutant and wild-type BTK. However, most of these agents are in the preclinical stage of development, and no clinical data exist at this time [21]. DD-03–171, a BTK degrader, has shown anti-proliferative activity in MCL cells in vitro and the patient-derived xenograft (PDX) model [29]. Other BTK-PROTAC, such as MT-802 (derived from an ibrutinib warhead lacking the meta-acrylamide moiety), and advanced agents with improved pharmacokinetic properties, such as SJF620 and L18I, have also been tested for their efficacy to degrade BTK [30–32]. UBX-382 is another novel BTK degrader that inhibits both wild-type and BTK mutant tumor growth in DLBCL via targeting the BCR pathway [33]. Though BTK-PROTAC has shown anti-proliferative activity and selective degradation of BTK in DLBCL, only a limited number of studies are available in MCL. Furthermore, these BTK degraders are yet to go into clinical trials, and clinical data on them is still largely lacking.

### BTK-independent BTKi resistance mechanism

Although non-covalent and covalent BTKis have been found to target BTK selectively, these inhibitors may not mitigate BTK-independent BTKi resistance mechanisms found in lymphoma. Below we discuss BTKi resistance mechanisms beyond their dependency on BTK status (expression or mutation).

#### Genetic cause of BTKi resistance in MCL

The mechanisms associated with genetic alterations leading to primary BTKi resistance include missense mutations or somatic DNA copy number alterations in high-risk genes, BCR signaling component alterations, mutations in DNA damage repair machinery, tumor suppressors, and NFkB pathway dysregulation. These genetic alterations are found to be present in MCLs at a higher rate than CLL at baseline, predisposing them to significantly higher treatment resistance. In search of BTKi resistance-associated gene expression signatures, Zhang et al. performed RNA sequencing of ibrutinib-sensitive and resistant MCL tumors and identified the differentially expressed genes involved in glycolysis, glutaminolysis, and mitochondrial biogenesis related to metabolic reprogramming of oxidative phosphorylation (OXPHOS)[34]. Importantly, activation of the OXPHOS pathway was identified as a driver of acquired ibrutinib resistance in MCL, and DNA methyl-transferase 3A (DNMT3A) was found to act as a mediator of mitochondrial biogenesis, which is required for OXPHOS activation in MCL. Considering genetic alterations beyond *BTK*^*C481S*^ mutation as the primary cause, Rahal et al. demonstrated loss of function mutation in NF-κB inhibitors (*TRAF2, TRAF3*, and *BIRC3*) in ibrutinib-resistant MCL cell lines (*n* = 6) causing dependency of resistant cells on the MAP3K14 pathway which in turn activated the alternative NF-κB survival signaling. In contrast, ibrutinib-sensitive MCL cell lines (*n* = 4) displayed chronic activation of BCR signaling [35]. Furthermore, in the same study, mutations in *TRAF3* and *BIRC3* were further confirmed to be present in patients' tumors that did not respond to ibrutinib. In a phase-3 MCL3001 (RAY) trial (NCT01646021), Lenz et al. confirmed that primary ibrutinib resistance was associated with mutations in NF-κB inhibitor genes and the EGFR family of genes [36]. Loss of function mutation in *BIRC3* has also been demonstrated to activate non-canonical NF-κB signaling in MCL, which is quite different from BCR-dependent classical NF-κB activation signaling [37]. Using 13 paired primary MCL tumors and a whole exome sequencing approach (WES), Chenglin et al. identified 18 recurrently mutated genes, including *ATM, MEF2B,* and *MLL2* and novel mutation targets such as *SIPR1* and *CARD11*. *CARD11* is a scaffold protein that acts downstream of BTK signaling and functions by regulating NF-κB signaling. Investigation of 173 MCL samples identified that 5.5% of MCL cases harbor *CARD11* mutation [38]. Genomic profiling of 24 MCL patients (phase 2 clinical trial; NCT02471391) who received ibrutinib treatment for four weeks, followed by venetoclax (a BCL2 inhibitor), identified genetic signatures between responders and non-responders. Five of 24 MCL patients did not respond to treatment and had 9p21.1–p24.3 loss and mutation in *SMARCA2* genomic region (4/5) or deletions in *ARID2* (3/5).

Furthermore, a mutation in the *ATM* gene was observed in patients who achieved CR [39]. Large-scale genomic data has provided the landscape of somatic mutations and clonal evolution of hotspot mutations in *CCND1* (E36K, Y44D, and C47S) (cyclin D1 gene), leading to the accumulation of the cyclin D1 protein through its defective proteolysis [40]. Importantly, it was found that these *CCND1* mutations are associated with ibrutinib resistance in MCL [34, 41].

Naeem et al. showed genetic causes beyond *BTK*^*C481S*^ mutation as responsible for BTKi resistance in CLL. WES on primary CLL patients who experienced disease progression following pirtobrutinib treatment revealed the presence of a second-site BTK mutation (T474I) [42]. The primary cause of such BTKi resistance-associated genetic alterations could be intratumoral heterogeneity present at the time of diagnosis and modulation of the initial mutational profile at the progression of the disease [40, 43].

#### Cancer stem cells and BTKi resistance in MCL

Besides genetic causes, non-genetic molecular changes have been found to cause the development of intrinsic and acquired BTKi resistance. Other non-genetic mechanisms, such as PI3K-AKT-mTOR, non-canonical NFκB activation, or epigenetic gene dysfunctions that bypass the survival BCR signaling may also contribute to BTKi resistance. Modulation in tumor immune microenvironment (TIME) and the presence of cancer stem cells (CSCs) have also been found to be associated with BTKi resistance development [13, 21, 44]. Chen et al. identified the CD19-/CD45 + cellular population in primary MCL tumors with CSCs-like characteristics (high expression of the stem cell-specific genes *Oct4* and *Nanog*) and were quiescent. Interestingly, even CD19-/CD45 + cells could form a complete heterogeneous tumor in immunocompromised mice compared to CD19+/CD45+ MCL cells [45]. Subsequently, Mathur et al. identified Wnt signaling as a critical oncogenic pathway activated in MCL CD19-/CD45+ CSCs cells (also called MCL-initiating cells or MCL-IC cells) and associated with ibrutinib resistance [46]. Interestingly, the mechanism of how CD19+ non-IC becomes IC is still largely unknown; interrogating the underlying mechanisms can identify rationale therapeutic targets. Though CD19-/CD45+ cells have been found to represent CSC characteristics in MCL tumors, no such molecular features of CSCs have been identified in DLBCL tumors [47], possibly due to the different histological makeup.

#### Tumor microenvironment and BTKi resistance

TME has been found to facilitate tumor cell growth by providing bi-directional signaling between tumor cells and the stromal compartment, including cellular and soluble factors. The cellular milieu in TME includes mesenchymal stromal cells (MSCs), fibroblasts, endothelial cells, immune cells (Treg, NK cells, macrophages), and the soluble component includes cytokines and growth factors. A study by Medina et al. has shown that bone marrow-derived MSCs secrete B cell activating factor (BAFF) and protect MCL cells from BTK inhibition via activating canonical and non-canonical NFκB pathways [48]. A report from Zao et al. has identified that the interaction of TME and MCL cells can induce innate and acquired resistance to BTKis via activating the PI3K-mTOR pathway and integrin-β1 signaling [15]. Other reports have identified the importance of the PI3K pathway and integrin-VLA-4 signaling in facilitating BTKi resistance or enhanced focal adhesion kinase (FAK) or CXCR4 activity in MCL during MCL–stromal cell interaction [49–51]. Evasion of the immune system is also a potential resistance mechanism for BTKis. MCL cells can evade the immune system by downregulating the expression of surface antigens or by developing a hostile TME that inhibits the function of immune cells such as T cells. Balsas and colleagues directly linked SOX11 expression to immunosuppressive microenvironment characteristics in MCLs, including reduced expression of antigen presentation gene, T cell activation factors, or increased Treg cell infiltration in TME. As such, this immunosuppressive microenvironment could potentially be associated with BTKi resistance development in MCLs [52].

### Targeting BTKi-resistant mechanism in MCL: beyond BTKi

Historically, MCL patients who progress after BTKi treatment (e.g., post-treatment with FDA-approved ibrutinib) have been found to have poor outcomes. Recently approved BTK-targeting non-covalent inhibitor pirtobrutinib has shown promising outcomes in R/R MCLs in BRUIN Phase 1/2 trial (NCT03740529) who progress on covalent BTKi. However, resistance development via activation of BTK-independent mechanism as mentioned earlier remains a significant hurdle toward the development of BTKi in MCL therapy. Therefore, therapies targeting beyond BTK are required for BTKi-resistant MCL tumors. In that regard, many small molecule inhibitors that target different oncogenes, some of whom have already received FDA approval for MCL treatment, are discussed and summarized in Table 2.

**Table 2:** Active agents and combinations in clinical trial history for BTKi-resistant MCL

Drug or combination	Target inhibitor	Study (N)	Outcomes	Adverse events	CT identifier (Ref)
MK-1026	BTKi	R/R Phase 1 (6)	ORR 57.9%	Fatigue (33%), constipation (31%), dysgeusia (28%), cough (25%), nausea (25%), pyrexia (25%), dizziness (23%), hypertension (23%), peripheral edema (22%), diarrhea (21%), and arthralgia (20%)	NCT03162536
Bortezomib	Proteosome	R/R Phase 2 (155)	DOR 9.2 months OS 13.4 months	Grade 3 neuropathy, Thrombocytopenia	NCT00063713 [53]
Venetoclax	BCL2	R/R Phase 1 (28)	ORR 67% PFS 11.3 months DOR 15.7 months	Neutropenia (19%), anemia (17%), and thrombocytopenia (15%)	NCT01328626 [54]
Ibrutinib + Venetoclax	BTKi BCL2	R/R Phase 2 (24)	PFS 29 months OS 32 months	Infections, Grade 1-2 nausea, and diarrhea	NCT02471391
Idelalisib	PI3Kδ	R/R Phase 1 (40)	ORR 40% DOR 2.7 months	Diarrhea (40.0%), nausea (32.5%), pyrexia (27.5%), fatigue (25%), rash (22.5%),	NCT00710528 [55]
Parsaclisib	PI3Kδ	R/R Phase 2 (108)	ORR 70% DOR 13.7 months PFS 11.99 months	Diarrhea (34.3%), pyrexia (17.6%), and constipation (13.0%)	NCT03235544
Idelalisib	PI3Kδ	Phase 1 (40)	ORR 40% CR 5% PR 35%	Diarrhea (40.0%), nausea (32.5%), pyrexia (27.5%), fatigue (25%), rash (22.5%)	NCT00710528 [55]
Lenalidomide	Immune Modulator	R/R Phase 2 (134)	ORR 28% DOR 16.6 months PFS 4 months OS 19 months	Neutropenia (43%), thrombocytopenia (28%), anemia (11%), pneumonia (8%), and fatigue (7%)	NCT00737529 [56]
Lenalidomide vs (cytarabine, rituximab, gemcitabine, fludarabine, or chlorambucil) SPRINT	Immune modulator	R/R Phase 2 (254)	ORR 68% D0R 16.8 months	Neutropenia 44%, thrombocytopenia 18%	NCT00875667 [57]
Lenalidomide	Immune modulator	R/R Phase 2 (58)	ORR 29% CR 14% DOR 20 months	Peripheral edema 19% Fatigue 38%	NCT02341781 [58]
Pembrolizumab	Immune checkpoint inhibitor	Phase 1/2 (12)	ORR 25% CR 8.3% PR 16%	Grade 3 neutropenia	NCT02650999 [59]
Varlilumab (CDX-1127)	CD27mAb	Phase 1 (35)	78% shrinkage of target lesions	Grade 1–2 fatigue, rash, nausea, and diarrhea	NCT01460134
Daratumumab	CD38 mAb	Phase 2 (5)	ORR 6.7%, CR 13.3%	Cough, dyspnea, nausea, fatigue, anemia, Grade 3-4 neutropenia, and thrombocytopenia	NCT02413489 [60]
Vorinostat and Bortezomib	HDAC inhibitor + Proteasomal Inhibitor	Phase 1 (65)	PFS 7.6 months ORR 27%	Neutropenia and thrombocytopenia, Grade 3 gastrointestinal toxicity	NCT00703664 [61]
Vorinostat combined with RICE chemotherapy	HDAC	R/R Phase 1/2 (5)	ORR 60%	Grade 3 gastrointestinal toxicity, infection, hypokalemia, transaminitis	NCT00601718
Abexinostat	HDAC	R/R Phase 1/2 (11)	ORR 27.3% PFS 3.9 months	Grade 3 neutropenia and thrombocytopenia	NCT03939182 [62]
*Palbociclib*					
(PD-0332991)	CDK4/6	R/R Phase 1 (17)	ORR 18% PFS > 1 year	Grade 3–4 neutropenia and thrombocytopenia	NCT00420056 [63]
Palbociclib + Bortezomib	CDK4/6 + Proteasome	R/R Phase 1 (19)	ORR 27%	Neutropenia (63%) and thrombocytopenia (53%)	NCT01111188 [64]
Palbociclib + Ibrutinib	CDK4/6 + BTKi	R/R Phase 1 (27)	ORR 67% CDR 37%, 2-year PFS 59%	Neutropenia (41%) and thrombocytopenia (30%	NCT02159755 [65]
Abemaciclib	CDK	R/R Phase 1 (22)	ORR 23%	Neutropenia (32% grade ≥ 3), thrombocytopenia (32% grade ≥ 3), diarrhea (55%)	NCT01739309
AT7519M	CDK	R/R Phase 1 (12)	ORR 27%	Grade 3 gastrointestinal toxicity	NCT01652144
Flavopiridol	CDK	R/R Phase 1 (30)	ORR 11%	1 MCL patient with Grade 3 tumor lysis syndrome	NCT00058227
Everolimus (RAD001)	mTOR	Phase 2 (58)	ORR 8.6%, CR 0%	Anemia (20.7%), Thrombocytopenia (6.9%), Diarrhea (60.3%), Nausea (27.6%)	NCT00702052 [66]
VcR-CAP and R-CHOP (LYM-3002 trial)	Combination of BTKi	R/R Phase 3 (487)	ORR 64%	Grade 2 neutropenia and thrombocytopenia	NCT00722137 [67]
Venetoclax + Ibrutinib (AIM)	BCL2 + BTKi	R/R Phase 2 (23)	ORR 71%	Neutropenia and thrombocytopenia	NCT02471391
Venetoclax + ibrutinib (SYMPATICO)	BCL2 + BTKi	R/R Phase 3 (352)	ORR 81% CR 31%	Diarrhea 83%, fatigue 75%, and nausea 71%	NCT03112174 [68]
ABT-199 + Ibrutinib	BCL2 + BTKi	R/R Phase 1 (37)	ORR 83% CR 42%	Grade 4 neutropenia, Grade 3 diarrhea, Grade 3 respiratory disorder	NCT02419560 [69]
Rituximab + Ibrutinib	CD20 mAb + BTKi	R/R Phase 2 (113)	ORR 88% CR 44%	Atrial fibrillation 10%	NCT01880567 [70]
Obinutuzumab (GUAGIN)	CD20 mAb	R/R Phase 2 (15)	ORR 27%	Infusion-related reactions (73%)	NCT00517530 [71]
Obinutuzumab + Ibrutinib (OAsIs)	CD20 mAb + BTKi	Chemo naive + R/R Phase 1/2 (48)	CR 67% (Chemo naive) CR 86.6 (R/R) > 2 year PFS (69.5%)	Grade 3 thrombocytopenia and neutropenia	NCT02558816 [72]
Selinexor + Ibrutinib	Exportin-1 (XPO1) + BTKi	Phase 1 (3)	ORR 32%	Fatigue (56%), nausea (53%), anorexia (41%), and diarrhea (41%)	NCT02303392 [73]

*ORR* overall response rate, *PFS* progression-free survival, *CR* complete response, *PR* partial response, *DOR* duration of response, *CT* clinical trial

## Proteasome inhibitors

Bortezomib, a first-in-class compound that reversibly interacts with 20S proteasome, received FDA approval as a second-line therapy for R/R MCLs based on the outcomes of a phase-2 clinical trial (PINNACLE). In this trial, 155 MCL patients were treated with bortezomib monotherapy with a median duration of response (DOR) of 9.2 months [53]. Following updated time-to-event data in this clinical trial, a median OS of 23.5 months was achieved [74]. However, although bortezomib produced promising initial outcomes, more than half of the patients were refractory. The discovery of next-generation proteasome inhibitors, including carfilzomib and ixazomib, has not improved the treatment outcomes as expected [75]. Several resistance mechanisms associated with bortezomib have been reported in MCL, including activation of NFkB signaling, accumulation of anti-apoptotic protein Mcl-1, and accumulation of casein kinase 2 (CK2, the serine/threonine kinase and activator of STAT3) [76–78]. A report from Patricia et al. has shown that bortezomib resistance development is associated with plasmacytic differentiation of MCL cells with upregulation of interferon regulatory factor 4 (IRF4) and CD38 and CD138 expression [79]. Notably, overexpression of SOX11 in MCL was identified as a master regulator for the expression of IRF4 and PAX5 and was also found to block terminal B cell differentiation [80]. In addition, bortezomib treatment in MCL induces the transcription of a zinc finger protein (PRDM1, Blimp1) required for NOXA-induced apoptosis in MCLs and PRDM1 expression, which are critical for bortezomib efficacy in MCL [81].

## BCL2 inhibitors

BCL-2 is an anti-apoptotic protein frequently overexpressed in almost 90% of MCLs and identified to be amplified via 18q21 DNA copy gain locus leading to the overexpression of BCL2 [82]. Another study associated the high expression of BCL2 with the expression of LINK-A lncRNA in MCL [83]. Several BCL2-targeting agents have been discovered and are currently being investigated for MCL therapy. Venetoclax (ABT-199) is an oral BCL2-targeting agent that received FDA approval for CLL and SLL. Since MCL is also known to be a high BCL2 expression, the efficacy of venetoclax in MCL cases, either as a single agent or in combination therapies, has been investigated. In a phase-1 clinical trial without prior treatment, venetoclax as a single agent showed ORR in 75% of MCL (21/28) and CR in 21% of MCL (6/28) (NCT01328626) [54]. Another clinical trial using venetoclax as monotherapy on R/R MCLs (*n* = 28) and prior BTKi treatment showed ORR of 53% and CR of 18% [84]. Venetoclax has also been tested in high-risk MCLs that had progressed on BTKis or had multiple relapses on five prior therapies (*n* = 24). The ORR with venetoclax treatment was 50%, and a CR of 21% could be achieved [85]. Although venetoclax treatment as a single agent was effective in BTKi-resistant MCLs, other MCL cases found to be resistant to venetoclax have also been investigated. Patients who progressed on venetoclax treatment showed clonal evolution of genetic alterations of *SMARCA4* and *BCL2* and increased frequency of alterations in other genes (*TP53, CDKN2A, KMT2D, CELSR3, CCND1, NOTCH2,* and *ATM*) [85]. With a focus on synergistic relationships in MCLs, recent clinical trials seek to combine BCL2 inhibitors with BTKis or other targeting agents. In a multicenter retrospective cohort that evaluated the outcomes of patients with R/R MCL treated with venetoclax (*n* = 81), the authors reported an ORR of 40% with a median PFS of 3.7 months and OS of 12.5 months. In the study, 62% of the patients (*n* = 50) were treated with venetoclax monotherapy, 14% (*n* = 11) in combination with an anti-CD20 monoclonal antibody, and 20% (*n* = 16) with a BTKi or with other novel agents [86].

## PI3K-Akt-mTOR inhibitors

The phosphoinositide 3-kinase (PI3K) signaling pathway is often found to be crucially deregulated in ibrutinib-resistant MCL cells. Wang et al. developed an ibrutinib-resistant patient-derived xenograft (PDX) model of MCL (MCL-PDX) through chronic exposure to ibrutinib. They identified constitutive activation of PI3K-AKT-mTOR signaling as a crucial survival pathway in ibrutinib-resistant MCL cells leading to tumor development. Tumor growth of ibrutinib-resistant MCL-PDX was inhibited by combined treatment of PI3K-δ-targeting agent idelalisib plus ibrutinib [87]. Similar to these MCL studies, our group and others have also developed ibrutinib-resistant DLBCL cells and identified upregulation of PI3K-AKT signaling in resistant cells, which can be overcome by selective PI3K isoform inhibitors treatment [88, 89]. Five PI3K-specific isoform-targeting inhibitors had received FDA approval for hematological malignancies such as CLL/SLL and FL. These are copanlisib (p110α/δ), idelalisib (p110δ), umbralisib (p110δ), duvelisib (p110δ/γ), and alpelisib (p110α). In a phase 1/1b clinical trial, umbralisib in combination with ibrutinib (*n* = 21) showed an ORR of 67% and a CR rate of 19% (NCT02268851) [90]. Copanlisib in combination with ibrutinib demonstrated an ORR of 87.5% and a CR rate of 50% (NCT03877055) [91]. Buparlisib (pan-PI3Kδ inhibitor) in combination with ibrutinib (*n* = 18 with 17 evaluated for response) showed an ORR of 94% and a CR rate of 76% (NCT02756247) [92]. In a phase 1 clinical trial (NCT00710528), idelalisib showed an ORR of 40% with a DOR of 2.7 months [55]. Parsaclisib, a selective p110δ inhibitor, showed therapeutic potential (phase-II CITADEL-205 trials) in R/R MCL patients. The ORR was 70% in those who received parsaclisib without prior BTKi (*n* = 108) and 25% in those who received prior BTKi (*n* = 53) [93, 94]. Notably, it was also found that dynamic feedback interaction between MCL cells and stromal cells also contributes to ibrutinib resistance development and reciprocal activation of PI3K-AKT-mTOR as well as Integrin-B1 signaling, which could be reversed by combined disruption of BCR signaling with ibrutinib and PI3K-AKT-mTOR axis with GS-1101 (p110δ inhibitor) [15]. However, loss of PTEN or feedback amplification of other PI3K isoforms, such as p110α, has impaired the efficacy of idelalisib in MCL [95–97]. Therefore, inhibitors targeting the dual isoforms of PI3K have been generated and tested in MCL, including KA2237 (p110β/δ) [98].

## Immunomodulators

TME is one of the critical elements responsible for the R/R status of MCL. Immunomodulatory agents such as lenalidomide (Revlimid) directly influence tumor cells and various cellular compartments of the TME, including NK cells, stromal cells, and T cells, via activating antitumor immune responses [99, 100]. Lenalidomide exhibited antitumor activity in MCL cell lines by upregulating immune response genes, including CD40, CD58, and CD86, and inhibited IL6 production required for bone marrow-derived stromal cell activity [99, 101]. In 2013, lenalidomide was FDA-approved for R/R MCL based on results from MCL-001 (EMERGE; NCT00737529) and MCL-002 (SPRINT; NCT00875667) clinical trials for patients who were refractory to bortezomib treatment and were ineligible for intensive chemotherapy or stem cell transplantation. Lenalidomide-treated group had significantly improved progression-free survival (PFS) with a manageable safety profile [56, 57]. However, despite improved efficacy, lenalidomide treatment did not show promising results after BTKi treatment failure. In the MCL-004 trial (NCT02341781), MCL patients (*n* = 58) either relapsed, progressed, refractory, or intolerant to ibrutinib treatment had a cumulative ORR of 29% and CR of 14%, with a median DOR of 20 weeks. The ORR with lenalidomide monotherapy (*n* = 13) was 15% (*n* = 2), while the ORR with lenalidomide in combination with rituximab (*n* = 11) was 27% (*n* = 3) with CR rate of 9% (*n* = 1) [58]. The exact mechanism of lenalidomide resistance in BTKi-resistant MCL is unknown, but this could be attributed to the activation of PI3K-AKT signaling or other genetic alterations in MCL [102].

## Immune checkpoint inhibitors

Immune checkpoint proteins, including Programmed Death 1 (PD-1) and its ligands PD-L1 and PD-L2, Lymphocyte Activation Gene 3 (LAG-3), CD200, Cytotoxic T Lymphocyte Activator 4 (CTLA-4), and CD47, are involved in tumor immunology and benefit tumor growth. Except for T cells, other immune cells rarely express these immune effectors [103].

### CD47 and CD24

CD47 acts as a checkpoint that provides a "don't-eat-me" signal to macrophages via interaction with its surface protein SIRPα, resulting in immune evasion by the tumor cells. CD47 is overexpressed in cancer cells, which is the target of interest in MCL. Several monoclonal antibodies (Hu5F9-G4, AO-176, AK117, and CC-90002) and bispecific antibodies, including IBI322, PF-07257876 (target CD47 and PD-L1), IMM0306, CPO107 (targeting CD47 and CD20 at the same time), and TG-1801 (targeting both CD19 and CD47), are under investigation in a clinical trial for multiple lymphoid and solid tumor malignancies [104]. On the other hand, limited studies have focused on targeting CD47 in MCLs. So far, only three clinical trials are accruing patients, including a limited number of MCL cases (NCT04806035, NCT04599634, NCT05025800). A phase 1 clinical trial included 4 R/R MCL cases who received rituximab in combination with CD47-targeting ALX148 (decoy receptor fusion protein composed of SIRPα N-terminal D1 domain and been mutated for increasing its affinity for CD47 binding). However, ALX148 demonstrated excellent tolerability in MCL cases; only 2 out of 4 achieved partial response [105].

A recent study demonstrated that CD24 (a highly glycosylated cell adhesion protein–ligand; Siglec-10) expression but not the CD47 expression is associated with poor clinical response in MCL. Moreover, CD24 was also highly expressed in MCL cell lines where treatment of MCL cell lines with CD24-targeting antibody SN3 yielded 90% removal of MCL cells via phagocytosis by autologous macrophages. In addition, this study also identified that treatment with CD24-targeting antibody was superior to CD47-targeting antibody in MCL [106]. Many CD24-targeting agents, including monoclonal antibodies, chimeric antigen receptor (CAR) T cells, and bispecific antibodies, have been developed and tested in preclinical studies. Some are also in clinical trials for solid tumors [107, 108]. Studies about testing CD24-targeting agents' efficacy in MCL are minimal; therefore, more research is required in this area as MCL are also high CD24 expressers.

### PD-L1/PD-1

Similar to CD47 or CD24, limited data are available in MCL for another important immune checkpoint PD-L1/PD-1 expression and targeted therapies. In a study by Yang et al., the highest PD-L1 expression was observed in DLBCL, followed by SLL, mucosa-associated lymphoid tissue lymphoma, and MCL, and the lowest expression was found in FL [109]. Compared to normal PBMCs, Wang et al. 2013 described that the percentage of PD-L1-expressing cells is high in primary MCL tumors and most MCL cell lines. The authors also reported that this high PD-L1 expression in MCL inhibited the T cell activity and proliferation, impaired antigen-specific T cell responses, and rendered MCL cells resistant to T cell-mediated cytolysis. Additionally, the inactive phenotype of T cells due to high PD-L1 expression was reversed by blocking PD-L1 expression on MCL cells [110]. Harrington et al. also demonstrated the constitutive expression of PD-L1 in primary MCL cells, whereas expression of other immune checkpoint genes, including PD-L2, LAG-3, and CTLA-4, was absent. Mechanistically, it was identified that both IFNγ and CD40:CD40L interaction between MCL cells and activated T cells in a co-culture condition regulates PD-L1 expression in MCL cells, which was attenuated by concurrent treatment with BTK or PI3K inhibition [111]. Expression and activity of PD-L1 in MCL are controversial. In a recent report by Ameli et al., neither PD-1 nor its ligand PD-L1 are relevant targets for MCL treatment. Using 79 formalin-fixed paraffin-embedded blocks of MCL and immunohistochemistry of PD-L1, Ameli et al. showed that only 3.8% of MCL are positive for PD-L1 expression [103]. This limited expression of PD-L1 also contributes toward the partial response of PD-L1/PD-1-targeting agents in MCL. Data from 81 R/R lymphoma patients (including 4 MCL) treated with nivolumab (anti-PD-1-targeting antibody) as a single agent showed no significant clinical response in MCL. Three of four MCL patients experienced stable disease; surprisingly, these patients were negative for PD-L1 expression [112]. PD-L1-targeting drugs, as a single therapeutic agent, have inferior outcomes in MCL; therefore, clinical trials evaluate potential combination strategies in MCL. For instance, pembrolizumab (KEYTRUDA, anti-PD-1) plus ibrutinib in a phase-2/3 trial (NCT03153202) and nivolumab plus lenalidomide (NCT03015896) are under investigation for MCL and other subtypes of lymphoma.

### CD27

The other immune checkpoint proteins include CD27, a co-stimulatory molecule that negatively regulates T cell activation by engaging its ligand CD70. Notably, CD70 was identified as a direct target of the Sox11 gene and is overexpressed in SOX11^post^ MCL, not in Sox11^neg^ MCL. Sox11^post^ MCL is associated with an immune imbalance with increased effector Treg cells in the TME [52]. Varlilumab (CDX-1127) is a CD27-targeting monoclonal antibody that could reverse the T cell exhaustion status and is being under investigation in a phase-2 clinical trial in combination with nivolumab for aggressive B cell lymphomas, including MCL cases (NCT01460134) [113].

## Receptor tyrosine kinase-like orphan receptor 1

ROR1 was first identified to be overexpressed in CLL and then found to be overexpressed in several other lymphoma subtypes, including MCL [114, 115]. Importantly, ROR1 is absent in most normal adult tissues but overexpressed in other malignancies, required for tumor cell survival and metastasis, making it a suitable candidate for a therapeutic target [116, 117]. Many therapeutic agents, including small molecule inhibitors, CAR T cell products, and monoclonal antibodies targeting ROR1, have been developed and tested for their efficacy in MCL. The study from Ghaderi et al. identified that ROR1 is overexpressed in MCL cell lines and primary MCL tumors. Notably, treatment of MCL cells with ROR1-targeting small molecule inhibitor KAN0441571C inhibited ROR1 phosphorylation, non-canonical WNT signaling, and induced MCL cell death in a dose-dependent manner. In addition combining KAN0441571C with ibrutinib or other agents (venetoclax, idelalisib, everolimus, or bendamustine) showed a synergetic impact on MCL cell apoptosis [118]. Cirmtuzumab (UC-961), a humanized monoclonal antibody designed to inhibit ROR1 activity showed antitumor activity (inhibited MCL cell proliferation) in a preclinical model [119]. To enhance its antitumor activity, cirmtuzumab was conjugated to monomethyl auristatin E (MMAE) via a cleavable linker leading to the creation of zilovertamab vedotin (VLS-101 or MK-2140). Additional data pertaining to zilovertamab vedotin has been presented in the antibody–drug conjugate section below.

## Chromatin modifiers

Chromatin modifiers, including histone acetyl-transferase (HATs), histone deacetylase (HDACs), and DNA methyl-transferase (DNMTs), are deregulated in many B cell malignancies and displayed genome-wide DNA/histone modifications such as acetylation/de-acetylation, hypo/hyper-methylation, at the regulatory elements [120].

### HDAC inhibitors

Elevated expression of HDAC6 has been reported in B cell lymphoma compared to normal B cells, which is directly correlated to disease progression. Fimepinostat (CUDC-907) is a first-in-class oral small molecule inhibitor of HDAC and PI3K enzymes tested in MCL. A preclinical study using CUDC-907 as a targeting agent in primary MCL tumors and cell lines, including ibrutinib-resistant MCL-PDX model, demonstrated tumor regression and apoptosis of MCL cell lines via increasing histone acetylation in MCL [121]. Though this dual inhibitor has shown impressive anti-proliferative activity in ibrutinib-resistant MCL, for unknown reasons, this compound has not been tested in clinical trials for MCL (NCT01742988). Vorinostat (SAHA) is a 2^nd^ generation HDAC inhibitor tested as a single agent or combined with many other MCL treatment regimens. In a preclinical study of MCL, vorinostat as a single agent inhibited R/R/ MCL cell growth and induced apoptosis. Importantly this inhibitor showed synergistic anti-proliferative activity when combined with CDK4/6 dual inhibitor palbociclib [122]. A phase 1 trial of vorinostat and bortezomib had a modest activity for previously untreated (*n* = 22) and prior bortezomib treatment (*n* = 4) MCL with a median PFS of 7.6 months and 1.8 months, respectively [123], suggesting that vorinostat should be studied with a different combinatorial agent for treatment of R/R MCL. Multiple clinical trials tested vorinostat in combination with other agents for MCL including, rituximab, ifosfamide, carboplatin, and etoposide (NCT00601718) and cladribine and rituximab (NCT00764517) [61]. Abexinostat (formerly PCI-24781) is a new broad-spectrum hydroxamate-based HDAC inhibitor that affects chromatin organization and gene transcription in MCL and induces apoptosis in lymphoma cell lines in a caspase and reactive oxygen species-dependent mechanisms [124, 125]. Based on preclinical findings, abexinostat was evaluated in a phase 1/2 clinical trial (NCT03939182, *n* = 11) wherein it demonstrated an ORR of 27.3% with a median PFS of 3.9 months [62]. Given this, abexinostat is currently being studied with ibrutinib in MCL. Romidepsin and belinostat (PXD101) are also pan-HDAC inhibitors showing preclinical activity in MCL cell lines. Further combination of bortezomib with romidepsin and belinostat induced potent mitochondrial membrane depolarization and apoptosis in xenograft mice model; thus, this combination could offer a new sensible approach for treating MCL [126].

### PRMT5 inhibitors

Protein arginine methyl-transferase (PRMT5) is a type II arginine methyl-transferase that catalyzes the dimethylation of arginine residues on H3R8 and H4R3 of histone tails or other proteins. PRMT5 regulates multiple biological functions, including RNA processing, signal transduction, DNA damage response, and gene expression [127]. PRMT5 is overexpressed and dysregulated in MCL, including BTKi-resistant cells. Using ibrutinib-resistant MCL-PDX model, treatment with PRMT5 inhibitor PRT382 significantly reduced tumor burden and improved median survival in mouse models [128]. DNA damage repair genes such as ATM and TP53 are recurrently observed to be mutated in MCL, including CAR T cell therapies or those with intrinsic or acquired resistance to ibrutinib. Notably, ATM mutation in MCL led to the complete inactivation of ATM, which abrogated TP53 activation in response to DNA damage, allowing cells with unrepaired DNA to escape from TP53 surveillance [129, 130]. ATM mutated MCL cells are sensitive to PRMT5 inhibition, which was demonstrated using the ibrutinib-resistant MCL-PDX model. A recent report from Che et al. identified upregulated expression of PRMT5 in ibrutinib-resistant MCL tumors, which was associated with poor clinical outcomes. As PRMT5 is involved in epigenetic, post-transcriptional, and post-translational regulation of DNA damage response genes, PRMT5 inhibition by GSK3326595 induced downregulation of DNA damage genes (DNAPK, RAD51, NHEJ1) and induced oxidative stress markers leading to accumulation of DNA damage. Notably, in ATM-deficient MCL lines, PRMT5 inhibition by GSK3326595 resulted in more accumulated unrepaired DNA damage and attenuated MCL-PDX tumor growth. In addition, co-targeting MCL with PRMT5 inhibitor and ATR or CDK4 inhibitor had a synergistic response in both in vivo and in vitro MCL models [43]. A phase 1 clinical trial (NCT03886831) with PRT343 (a potent, selective, oral PRMT5 inhibitor) that included MCL and other malignancies with no available treatment options has completed accrual and awaiting read out. Many PRMT5 inhibitors, including GSK3326595, JNJ-64619178, and PRT811, have been developed, but most are being tested in other lymphoma subtypes or solid tumor malignancies [131]. Though PRMT5 inhibition emerged as an attractive therapeutic target in MCL, a recent study has also identified the development of primary or acquired resistance to PRMT5 inhibitors in MCL. PRMT5 inhibitor-resistant MCLs exhibited compensatory activation of multiple signaling pathways such as insulin receptors, PI3K, MAPK, and mTOR signaling in tumors, further using PRMT5 inhibitor (PRT-382) in combination with PI3K/mTORC1 and 2 (Omipalisib), or mTORC1 (Temsirolimus) or EIF1A (Silvestrol) could reverse this PRMT5 resistance in MCL [132].

### DNMT inhibitors

DNA methylation is an essential epigenetic mechanism in normal and cancerous cells that directly controls DNA regulatory elements and gene expression. Notably, variation in the magnitude of DNA methylation could be used as an independent prognostic factor for MCL prognosis, which probably could read the expression of essential tumor suppressors or oncogenes [133]. A study from Xin et al. identified upregulated expression of DNMT1 in primary MCL tumors, which was co-associated with the activation of the Wnt/β-catenin pathway. Treatment of MCL cells with arsenic trioxide, a DNMT inhibitor, downregulated Wnt/β-catenin target genes and DNMT1 expression [134]. Other DNMT inhibitors, including azacitidine and decitabine, have been FDA-approved for treating acute myeloid leukemia and myelodysplastic syndrome, but these agents have shown limited efficacy and toxicity in MCL and other B cell lymphomas [135]. Furthermore, DNMT3A was identified as a mediator of OXPHOS pathway activation via mitochondrial biogenesis and thus associated with ibrutinib resistance in MCL. Thus, targeting DNMT3A with a low dose of decitabine, which degrades DNMT3A protein, synergized with IM156, an inhibitor of the mitochondrial complex, could overcome ibrutinib resistance in MCL.

### Inhibitors of SUMOylation

SUMOylation is a post-translational modification of target proteins which is an essential step for the regulation of genomic integrity, gene expression, and intracellular signaling, which is deregulated in tumor cells. Selective inhibitor of SUMO-activating enzyme "Subasumstat" (TAK-981) identified to inhibit the growth of MCL cells when grown in stromal conditions and induced tumor regression in MCL-PDX model via inhibiting OXPHOS pathway and thus overcoming BTKi resistance mechanism. Furthermore, overexpression of the EGR1 gene was also identified to be upregulated in ibrutinib-resistant MCL cells associated with metabolic reprogramming and OXPHOS pathway deregulation in MCL, thus providing another strategy to target BTKi-resistant cells.

### EZH2 inhibitors

Like DNMTs, histone methyl-transferases such as EZH2 (enhancer of zeste homolog-2 inhibitors) have emerged as an attractive therapeutic target in B cell and other solid tumor malignancies [136]. Baquero et al. assessed the EZH2 expression in 166 primary MCL, where 57 cases (38%) were positive for EZH2 expression and were associated with aggressive histologic variants (65% vs. 29%), high Ki-67 proliferation rate (72% vs. 19%), and p53 overexpression (43% vs. 2%) compared to EZH2 negative tumors. Surprisingly, EZH2 expression was not correlated to the expression of other PRC2 components (EED and SUZ12) and H3K27me3, but this was associated with inferior survival outcomes in MCL [137]. Mutations in the SET domain of the EZH2 gene that increased its tri-methylation activity are prevalent in other B cell lymphoma but have not been reported in the case of MCL, suggesting that high EZH2 expression in MCL is sufficient for augmenting oncogenic signaling [40, 138]. Multiple EZH2-targeting inhibitors (GSK343 or GSK126, or OR-S1) are being tested in MCL those have shown significant anti-proliferative activity in in vitro and in vivo MCL-PDX models [139, 140]. An open-labeled multicentric arm phase-1 study identifying EZH2 inhibitor (XNW5004) efficacy in R/R B cell lymphoma, including MCL. Tazemetostat (EZM6438) is a potent orally bioavailable EZH2 inhibitor that initially received FDA approval for treating epithelioid sarcoma and R/R FL. Tazemetostat is now in phase-1 clinical trial for MCL (NCT03010982, NCT03028103). Another study identified Fibroblast Growth Factor Receptor-1 (FGFR1) as a significant candidate upregulated in relapsed MCL patients and cell lines when cultured under the influence of bone marrow stromal cells [141]. Moreover, the loss of FGFR1 abrogated EZH2 expression, improved survival in vivo [141, 142], and provided an alternative therapeutic strategy for targeting R/R MCL. EZH2 inhibitor tazemetostat in combination with zanubrutinib or ibrutinib in an ibrutinib/zanubrutinib-resistant MCL model showed synergistic activity in the MCL xenograft model [143].

## CDK4/6 inhibitors

Aberrant expression of cyclin D1 caused by a t(11;14)(q13;q32) chromosomal translocation is the hallmark of MCL. Cyclin D1 assembles CDK4/6 to phosphorylate retinoblastoma protein, releasing the E2F transcription factor to initiate oncogenic gene expression. As expected, the expression of cyclin D1 is significantly high in MCL cases compared to normal peripheral B cells. Expression of CDK4 but not CDK6 was elevated in MCL cells compared to peripheral B cells [144]. Three CDK4/6 inhibitors, palbociclib, abemaciclib, and ribociclib, received FDA approval for treatment of solid tumors [144]. Given the promising activity of CDK4/6 inhibitors in solid tumors and the high expression of cyclin D1/CDK4 in MCL, the efficacies of these agents were screened in clinical trials. In a phase-1 clinical trial (NCT00420056) by Leonard et al., CDK4/6 inhibitor palbociclib (PD0332991) was given to MCL patients (*n* = 17; 71% were at high/intermediate risk according to MCL International Prognostic Index score) that induced early G1 cells arrest and tumor regression in some patients. Five MCL patients achieved PFS time of > 1 years (18% ORR) with limited toxicities [63]. Given the modest clinical outcome achieved with palbociclib as a monotherapy, subsequent clinical trials were carried out of this agent in combination with other MCL-targeting agents. A phase-1 trial of palbociclib plus bortezomib was conducted (NCT01111188), including 19 MCL patients where an ORR of 24% (6% CR) with associated toxicities, including grade 3 neutropenia (63%) and thrombocytopenia (53%) [64]. A phase-1 trial was conducted where palbociclib was combined with ibrutinib (PALIBR) (NCT02159755). 27 MCL patients were treated with this combination; those appeared with improved outcomes (ORR of 67%, CDR of 37%, and 2-year PFS of 59%). The combination had an acceptable safety profile, including neutropenia (41%) and thrombocytopenia (30%), compared to previous palbociclib trials [65]. Other CDK inhibitors are also in clinical trials for MCL cases, including abemaciclib (22 R/R MCL, ORR of 23%), ribociclib (7 MCL, ORR 0%), AT7519M (12 MCL, ORR of 27%), and Flavopiridol (30 MCL, ORR of 11%) [144].

## Other therapeutic agents in MCL

Chemokine receptors and adhesion molecules like integrin are required for both regular and malignant B cells for trafficking and homing to supportive tissue microenvironments, including secondary lymph nodes. Stromal cells constitutively express chemokines, such as CXCL12, CXCL13, and many more, guiding B cell homing and positioning within the lymph node compartment [145]. However, very few studies have disseminated the expression and function of adhesion molecules in MCL. Kurtova et al. examined chemokine receptors, adhesion molecule expression, and their functions in MCL cells [146]. MCL cells expressing high levels of CXCR4 and CXCR5 chemokine and VLA-4 adhesion molecules are required for adhesion and spontaneous migration of MCL cells beneath the MSCs layer and are associated with drug resistance [146]. Further study by Chen et al. identified that silencing of CXCR4 expression in MCL significantly reduced proliferation and adhesion to bone marrow stromal cells. Moreover, co-culturing of MCL cells with either stromal cells or condition media from stromal cells prevented apoptosis of MCL after ibrutinib treatment, suggesting that interaction with bone marrow stromal cells some have protective effects on MCL from therapeutic agents [147]. As chemokine and adhesion molecules contribute to BTKi or other drug resistance in MCL, therapies targeting these molecules have been developed, and their antitumor efficacies in MCL have been tested. A recent report identified that CXCR4 expression is an independent poor prognostic factor for MCL and can be a promising target for imaging and radioligand therapy [148]. Plerixafor (CXCR4 antagonist) and natalizumab (anti-VLA-4 antibody) are the agents that could inhibit the interaction of MCL to stromal cells keeping these MCL cells in a mobilized state; these mobilized MCL cells are more susceptible to standard therapies [149].

Identifying alternative oncogenic pathways in BTKi-resistant MCL has been limited to protein-coding genes. However, one study identified subsets of miRNAs that regulate the MAPK-ERK cascade, including miRs-221, 146a, 182, 342, and the let-7 family members were downregulated in ibrutinib-resistant MCL cells, thereby causing upregulation of MAPK-ERK signaling which can be targetable by MEK inhibitor (cobimetinib) [150].

## Combination approaches with small molecule inhibitors to overcome BTKi resistance in MCL

While several agents targeting different MCL signaling as single agents have shown some activity, they are less effective in patients with BTKi-resistant MCL. Hence, combination approaches have now been studied to overcome resistance to BTKis in patients with R/R MCL. This section discusses various combination approaches for treating R/R MCL containing BTKis, or other agents tested in clinical trials.

Bortezomib has also shown anticancer activity in MCL preclinical model when combined with zanubrutinib [151]. A phase 1 clinical trial of bortezomib and ibrutinib has completed accrual in R/R MCL (NCT02356458). A phase 2 trial of bortezomib in combination with lenalidomide (CALGB 50501) only showed a modest ORR of 40% [152]. Combining bortezomib with rituximab, lenalidomide, and dexamethasone (DR2IVE) was well tolerated in ibrutinib-resistant MCL patients with an ORR of 100% and 3 out of 5 patients were still alive at the last follow-up [153]. However, this study was limited to a small number of MCL patients.

Ibrutinib in combination with venetoclax has been studied in three clinical trials in MCL. The AIM trial (NCT02471391) included 23 R/R MCL patients, with 50% exhibiting altered TP53 genes. Despite this, combining ibrutinib with venetoclax in this cohort showed an ORR of 71% [154], suggesting that the combination of ibrutinib and venetoclax-based treatment approach was highly active. In SYMPATICO (phase 3 trial enrolled 352 MCL patients, NCT03112174), the combination of venetoclax and ibrutinib demonstrated an ORR of 81% and CR of 31% at a median follow-up of 31 months [68]. In another study by Portell et al. ibrutinib 420 mg daily in combination with venetoclax at 200 mg daily (which is lower than the doses used in the AIM or SYMPATICO trials) provided comparable benefits with ORR of 83% and a CR rate of 42% (NCT02419560) [69]. The synergistic results of combining these two agents in clinical trials could be due to the mutual targeting of the common pathway by the respective agents. Regarding this, Li et al. identified that BTK expression was positively correlated with BCL2 expression, as targeting BTK by short hairpin RNA led to downregulating BCL2 and other anti-apoptotic gene expressions [155]. In addition, combining venetoclax and ibrutinib showed enhanced dephosphorylation of AKT or BTK and more PARP cleavage in MCL [156]. A BTKi, ibrutinib, or its 2^nd^ generation agents do not target mutant BTK. Therefore, BTK degraders have been synthesized, showing profound preclinical activity [21]. BTK degrader Nx-2127 also had synergistic anti-neoplastic activity when combined with BCL2 inhibitors at low doses.

CG-806 (luxeptinib) is a non-covalent kinase inhibitor targeting BCR-associated kinases LYN, SYK, and BTK, which are now under investigation in clinical trials [157]. CG-806 inhibited both wild-type and mutant BTKC^481S^. The study from Thieme et al. demonstrated the promising activity of CG-806 in the MCL-PDX model via disrupting BCR signaling networks [158]; thus, this agent can be a potential alternative molecule to be tested in MCL cases.

Bromodomain and extra-terminal (BET) family of proteins recognize the acetylated lysine on histone and regulate transcription of many oncogenes, including genes involved in the BCR pathway, BLNK, PAX5, Myc, and IKAROS family in MCL [159]. Notably, treatment with BRD4 inhibitor I-BET151 as a single agent inhibited MCL cell line proliferation in a dose-dependent manner [159]. Furthermore, bromodomain antagonist JQ1 has been tested in MCL cell lines, inhibiting the *MYC* gene and expression of NFkB target genes. In addition, JQ1 treatment showed a synergistic association in inducing apoptosis of the ibrutinib-resistant MCL cells when combined with another agent such as ibrutinib or panobinostat (pan-histone deacetylase inhibitor) or palbociclib (CDK4/6 inhibitor) or ABT-199 [160].

PI3Kδ Inhibitor zandelisib, combined with the BTKi zanubrutinib in patients with R/R MCL (*n* = 17), showed improved efficacy [161]. An ORR was 76% with a CR of 35%, the preliminary median PFS was 10.4 months, and very few patients discontinued the treatment due to adverse events reported [161].

## Combination approaches with monoclonal antibodies for the treatment of MCL

Rituximab was studied in combination with ibrutinib and was shown to be safe and tolerable in patients with R/R MCL. In an open-label, phase 2 trial in R/R MCL, rituximab in combination with ibrutinib resulted in higher responses with ORR and CR of 88% and 44%, respectively [70]. Rituximab was also studied in combination with venetoclax, and lenalidomide in the Nordic Lymphoma Group NLG-MCL7 (VALERIA) trial. This study included BTKi-resistant MCL and demonstrated an ORR of 40% (*n* = 6) with 4 patients achieving CR [162]. Rituximab combination with anti-CD74-targeting antibody milatuzumab has been tested successfully in the preclinical MCL models [163].

Obinutuzumab (GA101) is a CD20-targeting humanized antibody that has demonstrated efficacy in the MCL preclinical models [164]. This antibody has non-fucosylated sugars on the Fc portion and was designed to overcome mechanisms of resistance to rituximab. Obinutuzumab as monotherapy in R/R MCL (*n* = 15) showed ORR of 27% in the GAUGUIN phase-2 trial (NCT00517530) [71]. Subsequently obinutuzumab was studied in combination with ibrutinib and venetoclax in relapsed and untreated MCL patients (*n* = 48) (OAsIs; a phase-1/2 trial) (NCT02558816). This combination was well tolerated and showed CR of 67% in relapsed and 86.6% in untreated MCL patients [72]. This combination can be considered a possible salvage therapy for ibrutinib-resistant MCL patients [165]. However, despite high initial response rates in the OAsIs trial nearly 1/3 of patients relapsed [166]. In order to identify factors leading to resistance in the OAsIs trial, single-cell RNA sequencing and targeted DNA sequencing of patients' tumor samples were performed (*n* = 12 at baseline and *n* = 5 at relapse) that revealed a gain of function mutation in the *CARD11* gene [166]. Of note, while *CARD11*^mut^ tumor cells were minute (0.0005%) at the onset of treatment, all the cells carried a heterozygous mutation at the time of relapse. By integrating DNA sequencing data with single-cell RNA sequencing data, the authors identified the *CARD11* associated gain of function mutation named "OAsIs-R" signature that was also predictive for OS/PFS in MCL patients treated with conventional chemotherapy. Furthermore, BCL2A1 overexpression was identified as the top gene of the OAsIs-R signature, which can be targetable by MALT1 protease inhibition along with BCL2 inhibition in a synergistic fashion [166].

A third-generation CD20-targeting monoclonal antibody ublituximab (TG-1101) is a glycoengineered monoclonal antibody that has improved antibody-dependent cell-mediated cytotoxicity than rituximab. Ublituximab has been studied in combination with ibrutinib in R/R MCL (*n* = 15) and demonstrated an ORR of 87% with 33% CR [167].

## CAR T cell therapy in R/R MCL

CAR T cell therapy is an exciting new avenue for treating solid and liquid tumors, wherein host T cells are genetically engineered to express artificial receptors specific to target tumor-specific cell surface antigens.

### CD19-directed CAR T

CD19-directed CAR T cells have shown impressive outcomes in B cell lymphoma treatment when given either as monotherapy or in combination with other treatment regimens. Brexucabtagene autoleucel (Tecartus, KTE-X19) received FDA approval based on ZUMA-2 phase-2 trial for R/R MCL after chemotherapy and BTKi [168]. This study enrolled 74 R/R MCL patients where 62% of patients had primary BTKi resistance, 26% had a relapse after an initial response to BTKi therapy, 7% experienced relapse after stopping BTKi therapy, and 4% were intolerant of BTKi (had adverse events). The study had an ORR of 85%, with a CR of 59%. Adverse events include cytokine release syndrome (CRS) in 15% of MCL patients, cytopenias in 94%, and infections in 32% of cases [169]. A long-term follow-up (3 years) of the pivotal ZUMA-2 study of KTE-X19 has recently been reported. With a median follow-up of 35.6 months, the ORR for 68 treated patients was 91%, and CR was 68% (NCT02601313) [170]. These data, representing the most extended follow-up of CAR T cell therapy in patients with MCL, suggest that KTE-X19 induced durable long-term responses with safety profiles in patients with R/R MCL. Data from the Descar-T French registry (LYSA Group) has put forward the first results of KTE-X19 in R/R MCL who failed after at least one line of chemo-immunotherapy or BTKi treatment. 47 MCL patients were infused with the KTE-X19 CAR T product. The ORR was 88% with a CR of 61.9%, CRS was noted in 78.7% of patients, and neurotoxicity was observed in 48.9% [171]. Another real-world experience from the United States lymphoma CAR T consortium by Wang et al. presented data from 93 R/R MCL patients infused with the KTE-X19 CAR T product, where an ORR of 86% with 64% CR was achieved. CRS in 88% and neurotoxicity in 58% of patients were reported. [172] Real-world outcomes data of Brexucabtagene autoleucel [173], where 82 MCL patients R/R to BTKi were enrolled, showed a median follow-up of 9.1 months, ORR was 89.6% (83.3% CR, 6.3% PR).

Lisocabtagene maraleucel (JCAR017), modified to have 4-1BB as a co-stimulatory domain, has also been studied in 32 R/R MCL in the TRANSCEND NHL 001 phase-2 trial, and the ORR was 84%, including 59% CR [174]. CRS was noted in 50%, and neurologic events were present in 28% [174]. Ying et al. showed the efficacy and safety of Relmacabtagene autoleucel, a CD19-directed CAR T product in 11 R/R MCL in China [175]. Based on three months follow-up analysis, an ORR of 81% and CR of 54.5% with a low grade ≥ 3 CRS incidence were noted. A recent study on AUTO1 CD19-targeting CAR T cells (designed to reduce toxicity and improves engraftment) showed a 100% ORR rate in MCL (*n* = 3). A large cohort and multicentric study is required to evaluate its efficacy and toxicity further in R/R MCL patients [176]. A phase-1 single centric ENABLE clinical trial (NCT04049513) has been initiated using third-generation CD19 CAR (WZTL-002) incorporating the intracellular signaling domains of CD28 and Toll-like receptor 2 (TLR2) to identify a safety dose of R/R B cell NHL patients including MCL [177]. Other phase-1 study evaluating BAFFR-targeting CAR T cells in various B-NHL including MCL (NCT05370430).

### ROR1-targeting CAR T

Multiple therapeutic modalities have been developed to target ROR1 in hematological and solid tumor malignancies. PRGN-3007 UltraCAR-T is a first-in-class investigational multigenic, autologous CAR T cell therapy developed on Precigen's UltraCAR-T platform, which has been engineered to express a ROR1-targeting CAR receptor, a membrane-bound interleukin-15 (mbIL15) for enhanced in vivo expansion and persistence, a kill switch to conditionally eliminate CAR T cells for improved safety profile and intrinsic blockade of PD-1 gene expression. PRGN-3007 UltraCAR-T started its Phase-1 clinical trial for ROR1-positive hematological malignancies and solid tumors, including triple-negative breast cancer (NCT05694364). Preclinical studies using PRGN-3007 UltraCAR-T have so far shown a significant reduction in PD-1 expression, increased ROR-specific tumor cell cytotoxicity, and inflammatory cytokine production upon co-culture with ROR1 + PD-L1 + tumors, effectively reducing tumor burden in xenograft models with long-term persistence of PRGN-3007 UltraCAR-T in tumor-bearing mice [178].

### CD37-targeting CAR T

CD37 is a tetraspanin protein expressed in various B cell lymphomas, including MCL, and has been found to mediate tumor survival signaling [179]. GEN3009 is a CD37-targeting biparatopic antibody in a phase-1 clinical trial for multiple B cell lymphomas, including R/R MCL (NCT04358458). Besides therapeutic antibodies, CD37-targeting CAR T cells have also been designed into a third-generation lentiviral plasmid backbone with a CD8 hinge and 4-1BB co-stimulatory domain. CD37 CAR T cells have been preclinically tested for efficacy in B cell lymphomas which exhibited robust effector functions, Th1-type cytokines expression, and tumor clearance in the MCL-PDX model. Additionally, a bispecific CAR targeting CD19 and CD37 has also been developed to respond to either a single or both targets, and there was no discernible difference in cytotoxicity to CD19 or CD37 found [179]. Due to promising outcomes of CD37-targeting CAR T cells in preclinical in vitro and in vivo studies, a clinical trial exploring its efficacy in MCL cases will be the next logical step. Various other modified universal CAR products, such as WZTL-002 and SYNCAR-001, are under phase-1 clinical evaluations and are listed in Table 3.

**Table 3:** Cellular therapies and clinical trials for BTKi-resistant MCL

CAR therapy (Company)	Trial Name	Target	Study (N)	Outcomes	Adverse events	CT identifier (Ref)
Brexucabtagene autoleucel; KTE-X19 (Kite Pharma)	ZUMA-2	CD19	R/R MCL Phase 2 (74)	ORR 91% CR 68%	CRS 15%, cytopenias 94%, infection 36%	NCT02601313 [169, 170]
Brexucabtagene autoleucel; KTE-X19 (Kite Pharma)	US Lymphoma CAR T Consortium	CD19	R/R MCL Phase 2 (93)	ORR 86% CR 64%	CRS 88% ICANS 58%	NCT02601313 [172]
Lisocabtagene maraleucel; JCAR017 (Juno Therapeutics)	TRANSCEND NHL001	CD19	R/R MCL Phase 2 (32)	ORR 84% CR 59%	CRS 50% ICANS 28%	NCT02631044 [174]
Tisagenlecleucel (Novartis)	TARMAC	CD19	R/R MCL Phase 2 (20)	ORR 90% CR 80%	CRS 73%	NCT04234061 [180]
CD19 CAR T cells (Wuhan Union Hospital, China)	–	CD19	R/R MCL Phase 3 (24)	Recruiting	Recruiting	NCT05020392
CD19 CAR-CD28-CD3zeta-EGFRt-expressing Tn/mem-enriched T-lymphocytes (City of Hope Medical Center)	–	CD19	R/R MCL Phase 2 (36)	Recruiting	Recruiting	NCT04484012
WZTL-002 (Malaghan Institute of Medical Research)	ENABLE	CD19 with TLR2	MCL and other B-NHL Phase 1 (30)	Recruiting	Recruiting	NCT04049513 [177]
BAFFR-targeting CAR T (PeproMene Bio, Inc.)	–	BAFFR	MCL and other B-NHL Phase 1 (18)	Recruiting	Recruiting	NCT05370430
SYNCAR-001 (Synthekine)	–	CD19, co-expressing IL-2 beta receptor	MCL and other B-NHL Phase 1 (36)	Recruiting	Recruiting	NCT05665062
PRGN-3007 UltraCAR-T (Precigen, Inc Moffitt Cancer Center)	–	ROR-1	MCL and other B-NHL Phase 1/1b (88)	Recruiting	Recruiting	NCT05694364 [178]
RD14-01 (He Huang)	–	ROR-1	MCL and other B-NHL Phase 1 (18)	Recruiting	Recruiting	NCT05444322
LV20.19 (Medical College of Wisconsin)	–	CD19/20	Phase 1 (10)	ORR 100% CD 60%	Grade 1–2 CRS 10%	NCT03019055 [181]
MB-106 (Mustang Bio)	–	CD20	R/R MCL Phase 1 (3)	PR	No CRS or ICANS ≥ Grade 3	NCT03277729 NCT05360238
NKX019 (Nkarta Inc)	–	CD19	MCL and other B-NHL Phase 1 (150)	Recruiting	Recruiting	NCT05020678

*CAR* chimeric antigen receptor, *ORR* overall response rate, *NE* neurological events, *CRS* cytokine release syndrome, *CR* complete response, *ICANS* immune effector cell-associated neurotoxicity syndrome, *TLR2* Toll-like receptor 2, *B-NHL* B cell non-Hodgkin’s lymphoma

## Combination strategies with or after CAR T cell therapy

Despite the impressive outcomes of CAR T cell therapy, limitations such as toxicities (CRS and neurological), unavailability of robust CAR T cell expansion, failed engraftments, and resistance to CAR T cells have come up as potential hindrances to its growth [182]. These limitations can be overcome to a certain extent by prolonging BTKi treatment (≥ 5 cycles) before collection of autologous T cells or combining BTKi along with CAR T cell therapy which could reduce the immunosuppression markers (PD-1) on CAR T cells, prolonging the duration of remission [183, 184]. A preclinical study using MCL cell lines and mice xenograft model showed that combining ibrutinib with CD19 CAR T cell provided long-term remission of 80–100%, compared to 0–20% for the CAR T cell therapy-only treatment group [185]. The efficacy of ibrutinib and tisagenlecleucel (anti-CD19 CAR T product) was explored in confirmed BTKi-resistant MCL (*n* = 21) [186]. Twenty patients (44% of patients had a mutation in the TP53 gene) were infused with the CAR T product, and 75% of patients experienced CRS; 11/15 (73%) grade 1–2 with ORR at 13 months was 90% with CR of 80% [186]. To understand the oncogenic axis of dual-resistance to BTKi and CAR T therapy axis, Jiang et al. performed single-cell sequencing using 39 longitudinal samples from 15 MCL patients sequentially treated with BTKi and CAR T cell therapy [187]. Further bioinformatics and functional analysis identified that the HSP90-MYC-CDK9 axis was associated with dual-resistance development. Treatment with an HSP90 inhibitor (PU-H71 or 17-AAG) and a combination of CDK9 inhibitors (AZD4573) induced impressive anti-MCL activity both in vitro and *in vivo* the MCL-PDX model [187]. Using a xenograft model, the antitumor activity of LP-284 (a novel DNA-damaging agent) was evaluated in MCL, including those resistant to BTKi, bortezomib, or venetoclax [188]. LP-284 demonstrated antitumor activity with increased DNA damage in MCL cells [188]. A phase-2 clinical trial (NCT04484012) is underway to combine CD19 CAR with acalabrutinib in R/R MCLs.

Besides BTKis, BCL2-targeting agents combined with CAR T cell therapy have improved outcomes in preliminary and preclinical studies. However, these combinations have not yet been tested in clinical trials and are an avenue for further investigation for MCL treatment [189].

Identifying other B cell surface-specific markers or markers expressed by malignant B cell provided further development of new CAR T cell products that could reverse the previous CD19-directed CAR T cell therapy resistance (due to CD19 target loss) and other limitations [190]. Clinical trials are investigating the efficacy of CD20-targeting CAR T cells (NCT03277729).

Other CAR constructs include bispecific anti-CD19/CD20 CAR T cells (LV20.19) showed an ORR of 100% at 92 days with CR of 92% in phase-1 clinical trial of B cell lymphoma patients, including seven heavily pretreated MCL cases (NCT03019055) [181]. This data from NCT04186520, enrolling 10 MCL patients received LV20.19 CAR T cells, day 28 ORR was 100% (CR = 60% and PR = 40%) without any relapse at a median follow-up of 18 months and only 10% of patients had grade 1–2 CRS (no grade 3 + events was observed) [191]. Besides CAR T cell, modified CAR NK (NKX019), targeting CD19 is under clinical investigation for MCL (NCT05020678). Other NK cells-based CAR products including PCAR-119 or CAR.CD19-CD28-zeta-2A-iCasp9-IL15-transduced cord blood NK cells were enrolled in clinical trials, but recent data showed these therapeutic agents have been discontinued for unknown reasons.

## T cell engaging bispecific/trispecific antibodies

T cell-engaging bispecific antibodies are an emerging cancer immunotherapy class that has shown promise in treating several types of cancer, including MCL. These antibodies comprise two binding sites for different antigens: one recognizes the tumor-specific antigen, and the other binds to an epitope on T cells, typically CD3 receptor, which is required to bring T cells close to tumor cells and activate the T cell cytotoxic activity. The advantage of bispecific antibodies over monoclonal antibodies as therapeutic entities includes the direct cell-mediated killing of tumor cells via T cells, high affinity, and reduced treatment cost. There are many bispecific antibodies targeting B cell malignancies that are currently FDA-approved. Bispecific antibodies under clinical investigations in MCL are listed in Table 4. The familiar markers in all B cell malignancies are CD19 and CD20, and many bispecific antibodies targeting these antigens have been generated, and some have shown impressive activity against MCL in clinical trials.

**Table 4:** Bispecific antibodies in a clinical trial for BTKi-resistant MCL

Bispecific antibody (Company)	Trial Name	Target on T/B cells	Combinatorial agent	Study (N)	Outcomes	Adverse events	CT identifier (Ref)
Blinatumomab (Amgen Research)	MT103-104	CD3, CD19	–	R/R MCL Phase 1 (24)	ORR 71%	Grade 1–3 NE 22%	NCT00274742 [192]
NVG-111 (NovalGen Ltd.)	–	CD3 ROR-1	–	MCL and other B-NHL Phase 1 (90)	Recruiting	Recruiting	NCT04763083
Mosunetuzumab (Genentech, Inc.)	GO29781	CD3 CD20	Atezolizumab	R/R MCL Phase 2 (13)	ORR 30.8% CR 23%	Grade 3 Neutropenia	NCT02500407
Glofitamab (Hoffmann-La Roche)	GO41944	CD3 CD20	Obinutuzumab	R/R MCL Phase 1/2 (37)	ORR 83.8% CR 73%	Grade 1–2 NE 51.5% CRS 75.7%	NCT03075696
Glofitamab (The Lymphoma Academic Research Organisation)	–	CD3 CD20	Obinutuzumab	MCL and other B-NHL Phase 2 (78)	Recruiting	Recruiting	NCT04703686
Epcoritamab (Genmab)	EPCORE NHL-1 trial	CD3 CD20	–	R/R MCL Phase 1/2 (4)	ORR 50% CR 25%	CRS (49.7%); grade 1 or 2: 47.1%; grade 3: 2.5%), pyrexia (23.6%), and fatigue (22.9%)	NCT03625037 [193]
Odronextamab (Regeneron Pharmaceuticals)	ELM-1	CD3 CD20	–	R/R MCL Phase 1/2 (78)	ORR 57.9%	Anemia and lymphopenia	NCT02290951 [194]
PSB202 (Qilu Puget Sound Biotherapeutics)	–	CD20 CD37	–	MCL and other B-NHL Phase 1 (110)	Recruiting	Recruiting	NCT05003141
IGM-2323 (IGM Biosciences, Inc)	–	CD3 CD20	–	MCL and other B-NHL Phase 1/2 (260)	Recruiting	Recruiting	NCT04082936
GEN3009 (Genmab)	–	CD37 CD3 CD20	Epcoritamab	MCL and other B-NHL Phase 1/2 (182)	Recruiting	Recruiting	NCT04358458
Plamotamab (Xencor, Inc.)	–	CD3 CD20	–	MCL and other B-NHL Phase 1/2 (182)	Recruiting	Recruiting	NCT02924402

*ORR* overall response rate, *NE* neurological events, *CRS* cytokine release syndrome, *CR* complete response, *B-NHL* B cell non-Hodgkin’s lymphoma

### CD19/CD3-targeting bispecific antibody

Blinatumomab (Blincyto, AMG103, MT103) is a CD19-targeting bispecific antibody widely investigated in B cell lymphoma, including MCL. In a phase-1 clinical trial with 24 R/R MCL patients, blinatumomab, when given as a single agent, resulted in an ORR of 71% [192] in R/R MCL, which was higher than the ORR response achieved in DLBCL (55%). In the long-term follow-up studies including 13 MCL patients, the median OS was 4.6 years, PFS was 6.7 months, and the treatment-free survival (TFS) was 7.6 months [195]. Another bispecific antibody NVG-111 is a T cell engager with CD3 binding arm for T cells while simultaneously targeting ROR1-expressing malignant cells. NVG-111 is currently under evaluation in a phase-1 trial for B cell malignancies, including R/R MCL (NCT04763083).

### CD20/CD3-targeting bispecific antibody

CD20-targeting bispecific antibody mosunetuzumab (Lunsumio) has been evaluated for efficacy in a first-in-human phase-1/2 trial (NCT02500407) that includes 13 MCL patients. ORR of 30.8% (4/13), including 23% CR (3/13), was achieved [196]. Glofitamab (RG6026) is a 2:1 configuration bispecific antibody with a monovalent binding site for CD3 of T cells and bivalent binding to CD20 on B cells. A recent phase-1/2 clinical trial that included 37 R/R MCLs, 64.9% of whom had received prior BTKi treatment and 1000 mg or 2000 mg of glofitamab with Obinutuzumab showed ORR of 83.8% and 73% CR was achieved [197]. Glofitamab was tolerated well, with neurologic adverse events of grades 1–2 occurring in 19 patients (51.4%), and the most frequently reported adverse events were CRS in 75.7% of MCL patients [197]. There is another ongoing phase-2 clinical trial (NCT04703686) where glofitamab is evaluated for R/R B cell lymphoma, including MCL, for patients who have progressed on CAR T cell therapy. Epcoritamab (GEN3013) is a CD20-targeting and T cell engaging bispecific antibody which has been tested in multiple B cell lymphoma subtypes, including 4 MCL patients with responses observed in 50% (2/4) MCL patients and 25% was CR (1/4) (NCT03625037) [193]. Odronextamab (REGN1979) is a CD20/CD3-targeting bispecific antibody modified from an IgG4-base to reduce Fc binding. This is still under investigation in a clinical trial for MCL. Preliminary results for other heavily pretreated B cell lymphoma patients showed a durable response with an ORR of 57.9%. Other bispecific-based intervention recruiting in clinical trials for multiple B-NHL includes IGM-2323, engineered to contain ten high-affinity binding domains for CD20 and one binding domain for CD3 (NCT04082936). Plamotamab (XmAb13676) is another IgG1 bispecific anti-CD20/CD3 antibody in clinical trials under investigation for MCL (NCT02924402).

### CD20/CD37-targeting bispecific antibody

PSB202 is a novel anti-CD20/CD37-targeting antibody engineered by combining an Fc-enhanced humanized type II anti-CD20 IgG1 (PSB102) and a humanized anti-CD37 IgG1 (PSB107). PSB202 is in a phase-1 clinical trial for multiple B cell malignancies, including MCL cases (NCT05003141).

### Trispecific T cell activating antibodies

Besides bispecific antibodies, there have several trispecific T cell activating (TriTAC) antibodies been developed, such as HPN328 (DLL3 targeting), NM21-1480 (anti-PDL-1/anti-4-1BB/anti-HSA), HPN217 (BCMA targeting), but till date as per study suggest these TriTAC have been investigated in multiple solid tumors and multiple myeloma.

## Antibody–drug conjugates

Antibody–drug conjugates (ADCs) comprise a revolutionary cancer treatment strategy designed to target tumor cells more successfully and precisely when used alongside systemic cytotoxic chemotherapy. Monoclonal antibodies tailored for a tumor-associated antigen are combined with highly effective anticancer medicines (payloads or warheads) in their structure by a chemical linker. These antibodies can efficiently release cytotoxic payloads to tumor cells while sparing normal cells, lowering their unrequired toxicity.

### Zilovertamab vedotin

Zilovertamab vedotin (VLS-101 or MK-2140), a ROR1 ADC, was designed to improve the efficacy of ROR1-targeting antibody cirmtuzumab by linking it to MMAE via a cleavable linker. Zilovertamab vedotin had shown significant antitumor activity in MCL cell lines and in ex vivo primary patient samples. The ROR1-drug conjugate was also tested in previously treated MCL in a phase 1 trial (NCT03833180) that enrolled 15 MCL case with an ORR of 47% (4 PR and 3 CR) [198]. Zilovertamab vedotin was studied in combination with ibrutinib in a phase 1/2 clinical trial which enrolled 27 R/R and treatment naïve MCL patients. Zilovertamab was well tolerated and demonstrated an ORR of 85.2% (40.7% CR, 44.4% PR) with a median DOR of 34.1 months [199]. Zilovertamab vedotin in combination with venetoclax, induced almost total tumor regression in xenograft studies, showing the high combined cytotoxicity of these two drugs [200]. ROR1 is also significantly elevated in CD19-targeting CAR T relapsed MCL tumors. Importantly, in the PDX model, VLS-101 treatment significantly induced regression of MCL tumors resistant to ibrutinib, venetoclax, or CAR T cell therapy, suggesting that targeting ROR1 could be a feasible approach in the treatment of ROR1 positive MCL tumors, particularly those with failure to prior MCL therapies [201].

### Polatuzumab vedotin

Polatuzumab vedotin is an ADC-targeting cell surface receptor CD79B expressed by all B cell lymphomas. Polatuzumab vedotin received FDA approval based on study GO29365 (NCT02257567), in patients with R/R DLBCL. Polatuzumab vedotin-based combinatorial approaches are currently in clinical trials. These include polatuzumab vedotin + venetoclax, rituximab, and hyaluronidase (NCT04659044), polatuzumab vedotin + bendamustine and rituximab (NCT04913103), autologous stem cell transplant followed by polatuzumab vedotin ( NCT04491370), and polatuzumab vedotin and mosunetuzumab, a phase 1/2 study in patients with atleast 2 prior lines of systemic therapy in R/R MCL and other B-NHL (NCT03671018).

### Loncastuximab tesirine

Loncastuximab tesirine (ADCT-402) is an anti-CD19 monoclonal antibody that has been humanized and is attached to a toxin called pyrrolobenzodiazepine dimer. In the phase 1 with dose expansion study in R/R B-NHL, 183 patients were evaluable for assessment of the safety, clinical efficacy, drug kinetics, and immunogenicity of loncastuximab tesirine. Overall, 15 MCL patients were included with an ORR of 46.7% [202].

### Other ADCs

AGS67E, another ADC-targeting CD37, is currently undergoing clinical testing. In a phase I study in patients with R/R B- and T cell NHL (*n* = 2 MCL), the ORR was 22% [203]. Inotuzumab ozogamicin is a CD22-directed humanized monoclonal antibody conjugated to the cytotoxin, and calicheamicin. It was tested in combination with R-GDP (rituximab, gemcitabine dexamethasone cisplatin) in 13 MCL patients with three patients achieving CR and five PR [204]. SGN-CD70A targeting CD70 has also been studied in patients with R/R CD70-positive NHL (*n* = 20). Modest antitumor activity was noted in the study (1 CR and 3 PRs) and the applicability of SGN-CD70A was limited by the frequency and severity of thrombocytopenia [205]. There are no ADCs that are currently FDA-approved for treatment in R/R MCL. While the early data appears promising with some of the ADCs, additional research is required to further understand their effectiveness in patients with R/R MCL. Table 5 lists the important ADCs that are currently in clinical trials for treatment of R/R MCL.

**Table 5:** ADCs for BTKi-resistant MCL

Drug or Combination	Targets	Study (size)	Outcomes	Adverse events	CT identifier (Ref)
Zilovertamab vedotin (VLS 101)	ROR-1	R/R Phase 1 (15)	ORR 47%	Fatigue, Diarrhea, contusion	NCT03833180
Denintuzumab mafodotin (SGN-CD19A)	CD19	R/R Phase 1	No results posted	–	NCT01786135
Loncastuximab Tesirine (ADCT-402)	CD19	R/R Phase 1 (18)	ORR-47% CR-33.3% PR-13.3% PFS-4.8 months	Fatigue, edema, liver enzyme abnormalities	NCT02669017 [202]
Inotuzumab ozogamicin + R-GDP	CD22	R/R Phase 1 (13)	ORR-62%	Thrombocytopenia, neutropenia	NCT01055496 [204]
SGN-CD70A	CD70	R/R Phase 1 (5)	–	Thrombocytopenia, Fatigue, Anemia	NCT02216890 [205]
Polatuzumab vedotin + venetoclax + rituximab	CD79b	R/R Phase 2 (63)	Recruiting	–	NCT04659044
Polatuzumab vedotin + mosunetuzumab	CD79b and CD3/CD20	R/R Phase 1/2	Active, not recruiting	–	NCT03671018
AGS67E	CD37	Phase 1 (2)	ORR-22%	–	NCT02175433 [203]

*ADC* antibody–drug conjugate, *ORR* overall response rate, *CR* complete response, *R/R* relapse refractory, *R-GDP* rituximab, gemcitabine, dexamethasone, and cisplatin, *PR* partial response, *PFS* progression-free survival

## Conclusions and future direction

**Fig. 1:**
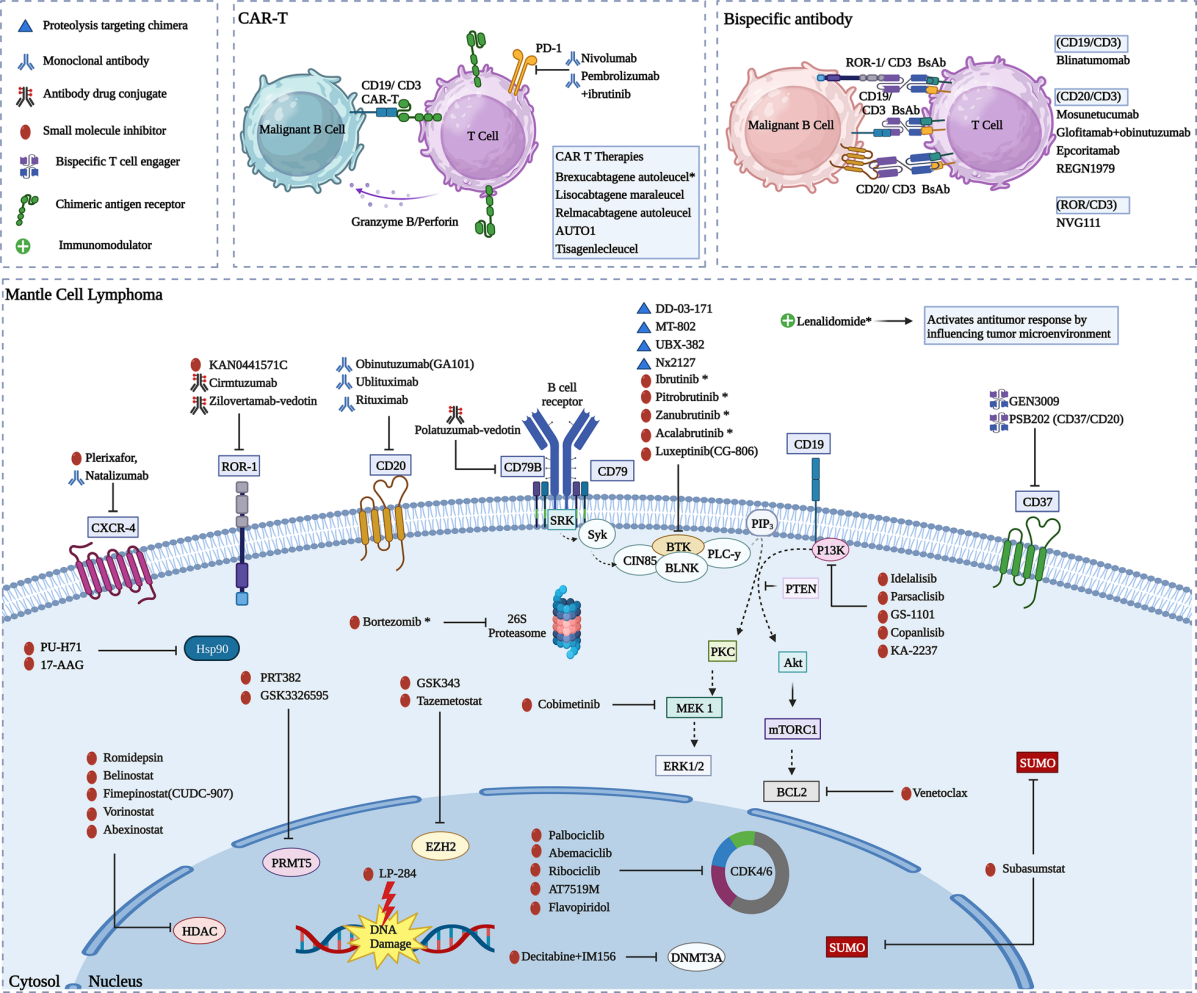
A summary of various MCL-targeting agents, including BTKi and other small molecular inhibitors, antibody–drug conjugates, chimeric antigen receptor T cells, bispecific antibodies, and other immune modulators

The advent of BTKi revolutionized the treatment landscape of MCL; however, the emergence of resistance and the poor outcomes for those progressing on BTKi remains a major concern. With a better understanding of the MCL biology, several small molecule inhibitors targeting the various BCR signaling pathways and ADCs have been developed (see Fig. 1) that have shown promising activity even in patients who are resistant or progressed on BTKi. In the last few years, CAR T cell therapies and bispecific antibodies have significantly changed the clinical trajectory with good outcomes even in high-risk MCL patients.

Although there are a plethora of therapies available for treating patients with BTKi-resistant MCL, sequencing of these agents remains a challenge. We envision a future where not all MCL patients receive the same therapies, in the same sequence, but rather where understanding tumor mutational profile, cellular pathways that drive proliferation, and the tumor immune microenvironment may inform treatment decisions paving the way for a *personalized treatment approach*.

## Data Availability

Not applicable.

## Abbreviations

MCL: Mantle cell lymphoma
CLL: Chronic lymphocytic leukemia
DLBCL: Diffuse large B cell lymphoma
FL: Follicular lymphoma
BTKi: Bruton's tyrosine kinase inhibitors
CAR: Chimeric antigen receptor
ADC: Antibody–drug conjugates
ORR: Overall response rate
PFS: Progression-free survival
CR: Complete response
PR: Partial response
DOR: Duration of response
CT: Clinical trial
NHL: Non-Hodgkin lymphoma
PDX: Patient-derived xenograft
PROTAC: Proteolysis-targeting chimera
TIME: Tumor immune microenvironment
TME: Tumor microenvironment
